# Supplementary material for: smalldisco, a pipeline for siRNA discovery and 3′ tail identification
Source: G3 (Bethesda). 2023 Apr 24;13(6):jkad092. doi: 10.1093/g3journal/jkad092 (PMC10234390; doi:10.1093/g3journal/jkad092)
Supplement: jkad092_Supplementary_Data [file jkad092_supplementary_data.zip › Supplemental_Material_Legends_G3-2023-404110.docx]

**Figure S1** Examples of siRNAs identified by smalldisco and segmentSeq that map to one region of the genome. Image is a screen shot from the Geneious genome browser. Green bars indicate the positions of transcribed genes, with the pointed end indicating direction of transcription. Yellow bars indicate coding exons. Gray bars indicate siRNAs as identified by smalldisco (top) and segmentSeq (bottom). Stacked black lines represent siRNA-derived read pileups.

**Table S1: List of NCBI accession numbers used in this study and frequency of siRNAs that map to exon-exon boundaries.**
