# Supplementary figures and images for: smalldisco, a pipeline for siRNA discovery and 3′ tail identification
Source: G3 (Bethesda). 2023 Apr 24;13(6):jkad092. doi: 10.1093/g3journal/jkad092 (PMC10234390; doi:10.1093/g3journal/jkad092)

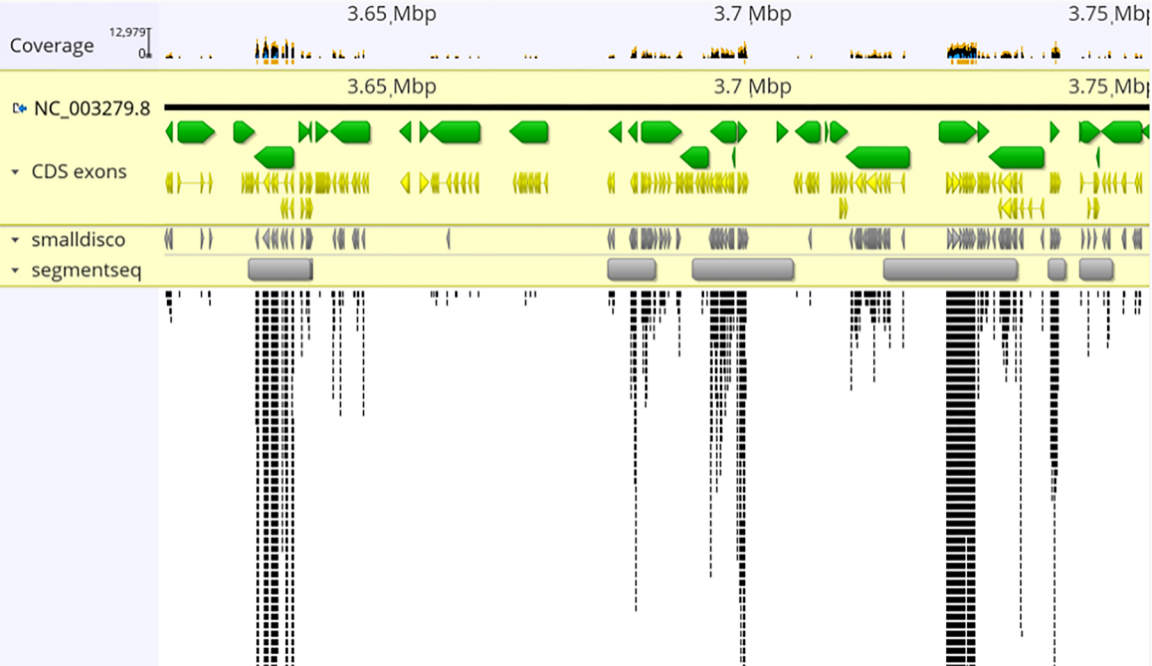

Supplement: jkad092_Supplementary_Data [file jkad092_supplementary_data.zip › Figure_S1_G3-2023-404110.pdf]
